# Experimental Investigation into the Mechanical Properties of Fine-Grained Tailings After Cemented Modification

**DOI:** 10.3390/ma18235380

**Published:** 2025-11-28

**Authors:** Yichen Wu, Guangjin Wang, Bing Zhao, Jun You, Songlin Li, Yuanting Zhu, Rong Lan, Mingsheng Liu, Qinglin Chen

**Affiliations:** 1 School of Land and Resources Engineering, Kunming University of Science and Technology, Kunming 650093, China; 2 School of Resources and Environmental Engineering, Jiangxi University of Science and Technology, Ganzhou 330044, China; 3 Guizhou Institute of Geo-Environment Monitoring, Guiyang 550081, China; 4 Department of Natural Resources of Guizhou Province, Guiyang 550081, China; 5 Kunming Engineering & Research Institute of Nonferrous Metallurgy Co., Ltd., Kunming 650051, China

**Keywords:** cemented modification, compressive strength, fine-grained tailings, flexural strength, multivariate nonlinear regression

## Abstract

Fine-grained tailings pose significant challenges for direct resource utilization applications such as tailings dam construction and backfill preparation due to their fine particle size, high specific surface area, and extended natural consolidation period. This investigation examined the mechanical properties of cemented fine-grained tailings under varying mix proportions and conditions. The cemented tailings were prepared using raw tailings material containing approximately 95% particles sized 0–74 μm. A comprehensive experimental program comprising 36 flexural tests and uniaxial compressive tests was conducted, with cement–sand ratio (A), curing age (B), and specimen immersion time (C) as controlled variables. The strength development mechanism was characterized through XRD and SEM, while mechanical performance data were systematically analyzed using range analysis, ANOVA, and regression analysis. Key findings demonstrate that ① the flexural strength of cemented tailings ranged from 0.43 to 2.07 MPa, with compressive strength varying between 3.02 and 12.52 MPa; ② both compressive and flexural strengths exhibited positive correlations with factors A and B, while showing negative correlation with factor C; ③ hydration products consisted primarily of C-S-H gels and zeolite-like phases, whose interwoven microstructure collectively ensured specimen integrity; ④ all three factors significantly influenced mechanical strengths with identical hierarchical impact: A > B > C; and ⑤ a comprehensive predictive model based on ternary quadratic polynomial regression was developed and validated. These results provide a scientific foundation for sustainable resource utilization of fine-grained tailings as solid waste materials.

## 1. Introduction

Tailings refer to the solid residues remaining after the extraction of valuable components from ores in mineral processing plants [1,2]. The continuous expansion of mining production has driven a corresponding increase in tailings output [3,4]. Currently, the predominant disposal method involves surface impoundment, where tailings dams are constructed to create storage areas for centralized containment [5,6]. However, these tailings dams not only occupy substantial land resources but also pose substantial environmental and safety risks [7,8]. Although typically discarded indiscriminately, tailings retain various components and possess significant potential for resource utilization [9,10]. Current approaches for comprehensive tailings utilization include: re-beneficiation of valuable constituents, production of agricultural materials, processing of construction materials, backfilling of underground mined-out areas, rehabilitation of open-pit mines, and land reclamation [11,12,13]. Among these applications, the development of fundamental engineering materials such as backfill for underground goafs and open-pit mines demonstrates particularly broad applicability, gradually emerging as a key research focus within the global scientific community [14,15,16].

Numerous research institutions and mining enterprises work in the field of comprehensive tailings utilization, achieving significant progress. Zhao et al. [17] investigated the physical and mechanical properties, failure characteristics, and early warning analysis of damage catastrophe in cemented tailings containing different initial defects. Jiang et al. [18] utilized a split Hopkinson pressure bar to study the fracture characteristics and energy consumption of cemented unclassified cemented tailings with different cement–sand ratios under impact loading. Jin et al. [19] conducted a series of tests on backfills composed of ultra-fine, fine, and coarse tailings to explore the influence of tailings particle size distribution on backfill strength and damage characteristics. Li et al. [20] analyzed the mechanical properties, strain field distribution, and microstructural characteristics of backfills prepared from tailings of different particle sizes, revealing the effects of tailings gradation, cement–sand ratio, and slurry concentration on the mechanical properties of backfill. Wang et al. [21] performed uniaxial compression tests on paste backfill materials with different moisture contents, finding that increased moisture content led to decreased compressive strength and elastic modulus, while the peak strain initially increased and then decreased. Estelle et al. [22] examined the effects of solid components—tailings, cementitious material, and water content—on the microstructural evolution and mechanical performance of uncemented unclassified cemented tailings mixtures. Aragón et al. [23] investigated the influence of tailings chemical composition, curing temperature, and specimen type on backfill performance, determining that uniaxial compressive strength peaked at a curing temperature of 40 °C. Siddique et al. [24] consolidated molybdenum tailings with cement, systematically testing the mechanical strength, elastic modulus, and chemical erosion resistance of backfills with different mix proportions to assess their engineering application feasibility. Shanmugasundaram et al. [25] conducted mechanical performance tests on subgrade materials incorporating varying amounts of magnesite tailings, evaluating their suitability as road subgrade material. Xiong et al. [26] prepared backfill using phosphate tailings as aggregate and optimized an XGBoost model with the WOA algorithm to predict the compressive strength of backfill. Fu et al. [27] employed phosphate tailings to fabricate lightweight ceramic materials via foam-gel casting, optimizing the stirring process to obtain high-strength porous structures and verifying their environmental safety. Yang et al. [28] combined experimental and simulation approaches to reveal the influence mechanisms of fine-grained tailings content on the mechanical properties of cemented backfill, systematically analyzing failure modes and energy transformation patterns.

Fine-grained tailings pose significant challenges in resource utilization due to their fine particle size, large specific surface area, and strong water retention capacity [29,30]. When used for tailings dam construction, fine-grained tailings exhibit slow natural sedimentation rates and extremely prolonged consolidation processes, making them prone to seepage instability and liquefaction potential during storage [31,32]. In engineering applications, these characteristics substantially impair the formation efficiency of internal skeleton structures and lead to insufficient cementitious reactivity [33,34]. Current research on tailings resource utilization primarily focuses on well-graded tailings, while systematic investigations into the mechanical properties of cemented fine-grained tailings under the synergistic effects of multiple factors—cement-sand ratio, curing age, specimen immersion time —remain scarce. This study employs fine-grained phosphate tailings with approximately 95% of particles below 74 μm as raw material and utilizes phosphate tailings-based cementitious material as cementitious material to prepare cemented tailings with different mix ratios (i.e., hardened mortar specimens of tailings-cementitious material mixtures). The research examines the mechanical characteristics and their variation patterns in cemented ultra-fine unclassified tailings, aiming to provide a scientific basis for the resource utilization of this solid waste material.

## 2. Materials and Methods

### 2.1. Experimental Materials

**Figure 1:**
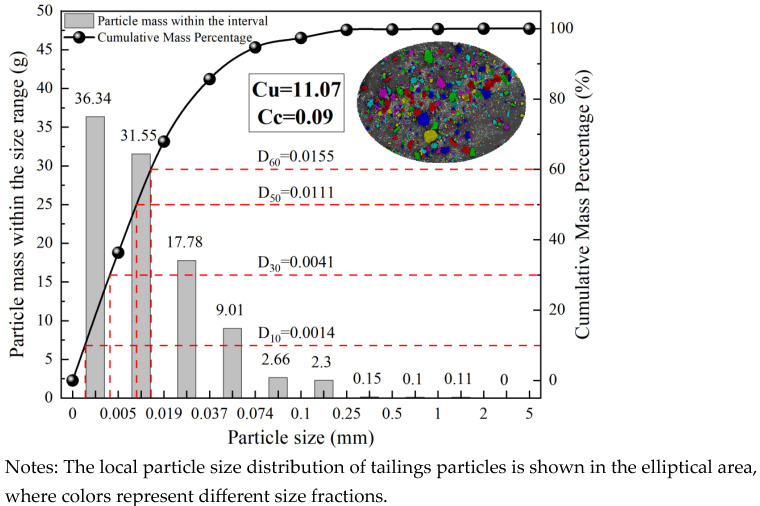
Particle size distribution curve of tailings.

**Figure 2:**
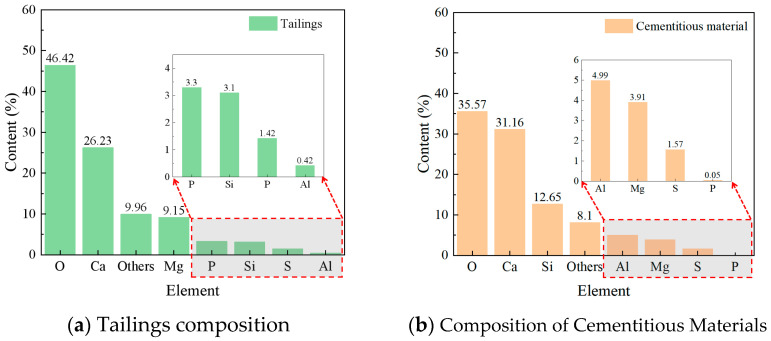
Elemental composition of tailings and cementitious materials.

The cemented tailings specimens were composed of three components: unclassified tailings, cementitious material, and water. The unclassified tailings were obtained from a phosphate mine in Yunnan Province, China. The cementitious material was a specialized cementitious material developed by a mining company, produced by grinding and mixing potentially reactive aluminosilicate materials—including blast furnace slag, fly ash, and phosphorus slag—with activating agents. Laboratory tap water was used throughout the experiments. Figure 1 presents the particle size distribution of the tailings, while Figure 2 shows the elemental composition analysis results of both the tailings and the cementitious material. Table 1 summarizes the physical parameters and chemical composition of the tailings and the cementitious material. Particle size analysis indicated that approximately 95% of the tailings particles were smaller than 74 μm, classifying the material as fine-grained tailings [35].

### 2.2. Experimental Design

To simulate the moisture conditions under coupled environmental factors such as rainfall erosion, leachate seepage, and groundwater immersion, the cement–sand ratio (A), curing age (B, days), and specimen immersion time (C, hours) were selected as the primary factors for treating cemented tailings specimens. All specimens were prepared at a fixed mass concentration of 75%. After reaching their designated curing ages, the specimens underwent either oven-drying or water immersion treatments.

The experimental design incorporated four cement–sand ratios (1:4, 1:6, 1:8, 1:10), three curing ages (7 days, 14 days, 28 days), and three specimen immersion times (0 h, 12 h, 24 h), resulting in a total of 4 × 3 × 3 = 36 distinct test condition combinations.

### 2.3. Experimental Procedure

**Figure 3:**
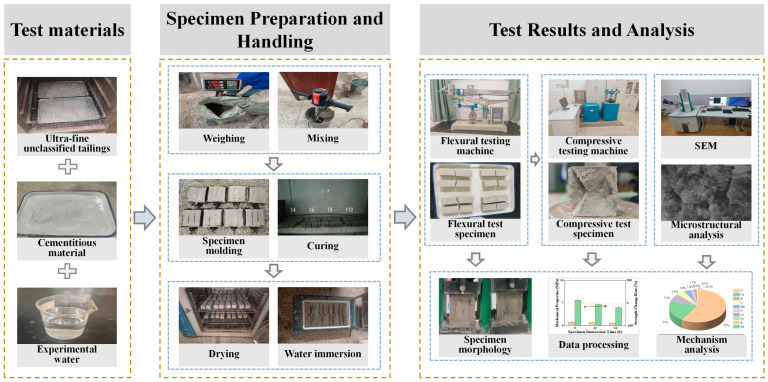
Experimental flowchart.

The tailings were first dried in an electric thermostatic drying oven at 80 °C for 24 h, then weighed and mixed with the cementitious material according to predetermined ratios. After adding water and stirring for 10 min, the mixture was cast into triple-gang prismatic molds measuring 40 mm × 40 mm × 160 mm. The specimens were rodded for compaction, struck off level, demolded, and subsequently placed in a curing room maintained under high-humidity conditions. Upon reaching their designated curing ages, compressive and flexural strength tests were conducted. Specimens demonstrating superior strength performance were selected for mechanism analysis. The experimental procedure is illustrated in Figure 3.

Flexural tests and compressive tests on cemented tailings were conducted in accordance with GB/T 17671-2021 [36]. For each test group, flexural tests were performed on three prismatic specimens, followed by compressive tests on the six resulting halves from the fractured specimens. The detailed testing procedure was as follows:

(1) Flexural test. The specimen is placed on a support cylinder on one side, and a load is applied vertically to the opposite face of the prism through a loading cylinder until the specimen undergoes brittle fracture, with the maximum failure load recorded automatically. The flexural strength is calculated using Formula (1):

(1) $$R_{f}=1.5LF_{f}/b^{3}$$

$R_{f}$ is flexural strength, MPa; L is the distance between support cylinders, mm; $F_{f}$ is the load applied to the midpoint of the prismatic specimen at failure, N; b is the side length of the prismatic specimen’s square cross-section, mm.

(2) Uniaxial Compressive Test. After completing the flexural strength test, two half-prismatic specimens are removed and placed in a cement compressive testing fixture. The load is applied at a constant rate until failure, and the maximum pressure is recorded. The compressive strength is calculated using Formula (2):

(2) $$R_{c}=F_{c}/A$$

$R_{c}$ denotes compressive strength, MPa; $F_{c}$ denotes maximum load at failure, N; *A* denotes compressed area, mm^2^.

## 3. Results and Analysis

The test results for the properties of cemented tailings are summarized in Table 2, which provides a complete dataset for subsequent analysis. Flexural strength characterizes the material’s resistance to bending failure, while compressive strength reflects its capacity to withstand compressive failure; both properties work synergistically to ensure structural stability and safety. As shown in Table 2, the cement–sand ratio, specimen moisture content, and curing age all influence the flexural and compressive strengths of the cemented tailings. The flexural strength of the cemented tailings ranges from 0.43 to 2.07 MPa, and the compressive strength ranges from 3.02 to 12.52 MPa. Both the flexural and compressive strengths reach their maximum values at a cement–sand ratio of 1:4, a curing age of 28 days, and a specimen immersion time of 0 h. 

### 3.1. Influence of Individual Factors on the Strength of Cemented Tailings

This study employed a controlled variable method for single-factor analysis. When examining the influence of a specific factor on the strength of the cemented tailings, the mechanical property data from all parallel tests at each level of that factor were averaged arithmetically. This average value served as the representative strength for that factor at the given level.

#### 3.1.1. Cement–Sand Ratio

**Figure 4:**
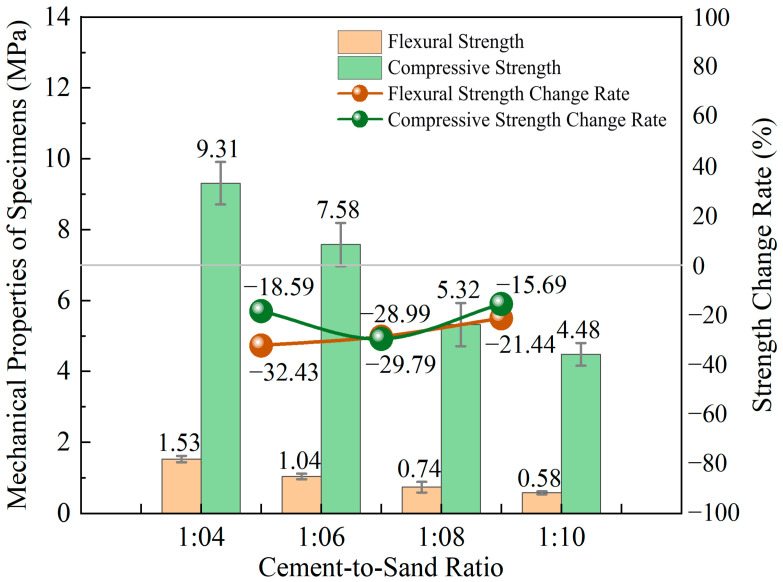
Column chart of strengths and line chart of variation rates for cemented tailings with different cement–sand ratios.

As the cement–sand ratio increased from 1:4 to 1:10, the flexural strength and compressive strength of the cemented tailings exhibited a decreasing trend. As shown in Figure 4, when the cement–sand ratio decreased from 1:4 to 1:6, 1:6 to 1:8, and 1:8 to 1:10, the average flexural strength decreased by 0.50 MPa, 0.30 MPa, and 0.16 MPa, respectively. Correspondingly, the average compressive strength decreased by 1.73 MPa, 2.26 MPa, and 0.83 MPa. All the strength change rates were negative, indicating that within the concentration range of this experiment, the strength of the cemented tailings was negatively correlated with the cement–sand ratio.

The cement–sand ratio directly governs the density and integrity of the cementation network within cemented tailings. At higher ratios, sufficient cementitious material thoroughly coats the tailings particles and participates in continuous hydration reactions, generating abundant hydration products that interweave to form a continuous, dense three-dimensional network. This significantly enhances both the compressive and flexural strength of the cemented tailings. Conversely, lower cement–sand ratios provide insufficient cementitious material to produce adequate hydration products for filling particle voids. This results in a discontinuous chemical cementation network, where particle cohesion relies primarily on physical interlocking rather than chemical bonding, consequently leading to reduced strength [37].

#### 3.1.2. Curing Age

**Figure 5:**
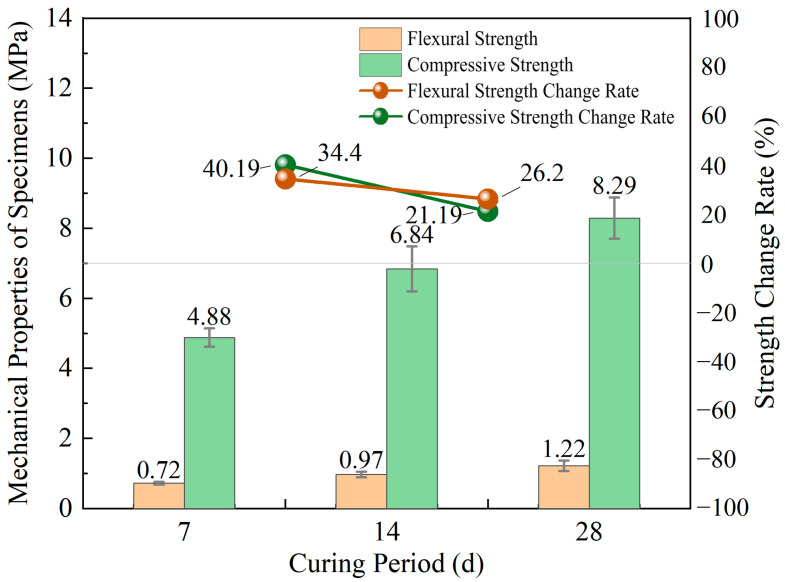
Column chart of strengths and line chart of variation rates for cemented tailings under different curing ages.

As the curing period extended from 7 days to 28 days, the flexural and compressive strengths of the cemented tailings showed an increasing trend. As shown in Figure 5, when the curing age increased from 7 to 14 days and further to 28 days, the average flexural strength consistently increased by 0.25 MPa at each stage, while the corresponding average compressive strength rose by 1.96 MPa and 1.45 MPa, respectively. The increase rates for flexural and compressive strengths from 7 to 14 days were 34.40% and 40.19%, respectively, while the rates from 14 to 28 days were 26.20% and 21.19%. The extended curing duration consistently enhanced both flexural and compressive strengths, although the rate of strength gain exhibited a gradually slowing trend.

The curing period governs the progression of hydration reactions in cementitious materials. During initial curing, rapid internal reactions generate substantial hydration products, leading to accelerated strength development. As curing age advances, the growing hydration products form a relatively dense microstructure that primarily fills larger pores [38]. Beyond a specific curing duration, active components become largely depleted while unreacted particles get encapsulated by dense hydration products. Concurrently, decreased concentration of reactive substances weakens the reaction driving force, resulting in markedly reduced strength growth rate. However, continued hydration of residual cement particles further fills internal voids, enhancing specimen density and strength [39]. This explains why Figure 5 shows a greater strength variation rate between 7 and 14 days than between 14 and 28 days in cemented tailings.

#### 3.1.3. Specimen Immersion Time

**Figure 6:**
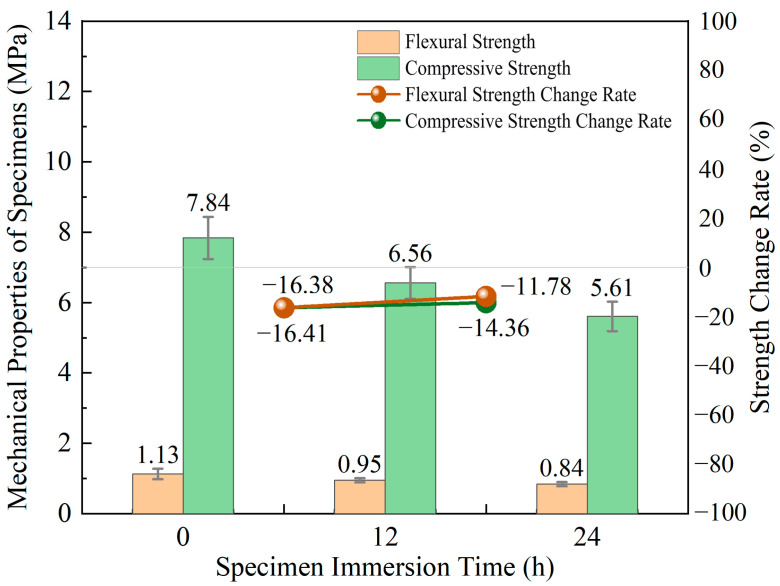
Column chart of strengths and line chart of variation rates for cemented tailings with different specimen immersion times.

As the specimen immersion time increased from 0 to 24 h, both the flexural and compressive strengths of the cemented tailings demonstrated a declining trend. As shown in Figure 6, within the selected standard group, elevated moisture content in the cemented tailings specimens corresponded with reduced flexural and compressive strengths. With prolonged immersion time, both strength parameters exhibited varying degrees of attenuation: the average flexural strength decreased by 0.19 MPa and 0.11 MPa, while the average compressive strength declined by 1.29 MPa and 0.94 MPa, respectively. The variation rates for both flexural and compressive strength registered negative values, confirming that increased specimen moisture content exerts an adverse effect on strength performance.

Based on the data calculated from Table 2, the average mass of the cemented tailings specimens was 448.80 g in the non-immersed state, increased to 491.36 g after 12 h of immersion, and reached 499.34 g after 24 h of immersion. The mass of the cemented tailings increased with prolonged immersion time, and the increment during the 0–12 h interval was significantly greater than that during the 12–24 h interval. During the initial immersion stage, the internal pore structure of the cemented tailings facilitated rapid water penetration and pore filling, resulting in a marked increase in mass. As immersion continued, most pores became occupied by water, and the dominant moisture transport mechanism gradually shifted to slow diffusion, leading to a reduced rate of mass gain.

As the specimen immersion time increases, water infiltrates the material’s pore network and micro-crack systems, significantly weakening the strength of the cemented tailings through both physical and chemical pathways. Extended water exposure enables moisture infiltration into the material’s pore network and micro-crack systems. Physically, water films develop at interfaces between cementation phases and tailings particles, weakening the physical adhesion between hydration products and aggregates. Simultaneously, moisture induces a lubricating effect between tailings particles, substantially diminishing interparticle frictional resistance [40]. Chemically, the immersion environment may trigger dissolution of hydration products from the cementitious materials. Water molecules penetrating into pores can disrupt cementitious phases through hydrolysis, resulting in structural softening, loosening, or even partial disintegration of the cementation network [41].

### 3.2. Post-Testing Specimen Appearance

#### 3.2.1. Fracture Surface Condition After Flexural Tests

**Figure 7:**
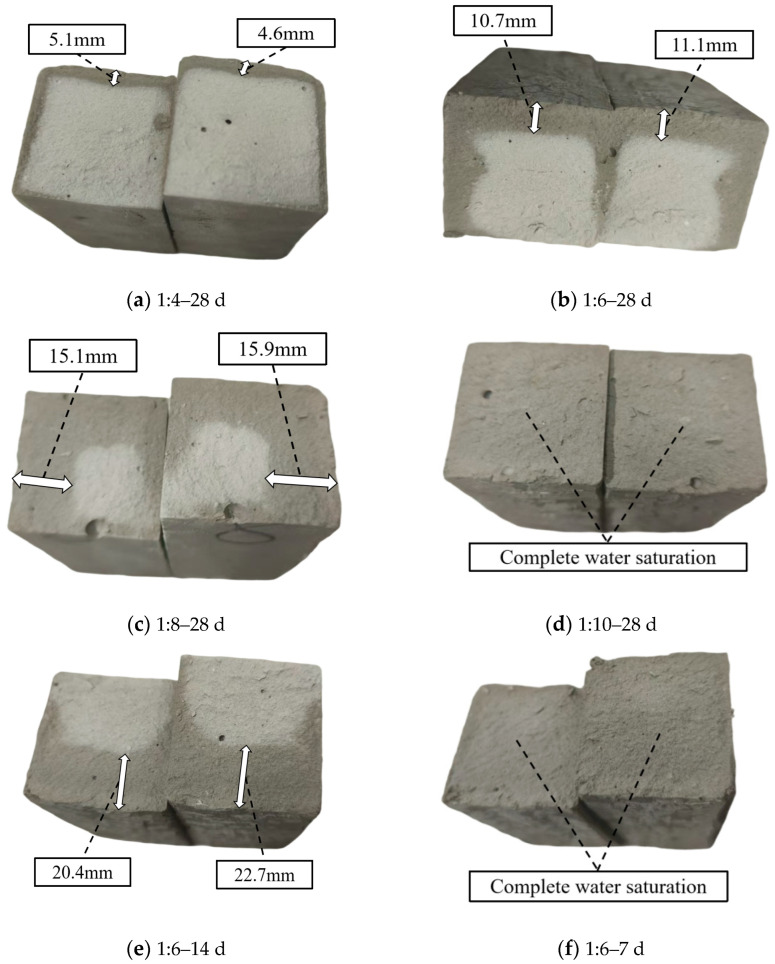
Water penetration on fracture surfaces of specimens after flexural testing (24 h immersion).

The fracture surface, as a directly exposed internal structure, enables the analysis of internal pore characteristics (such as porosity, pore size distribution, and connectivity) by observing and comparing the moisture penetration patterns on newly formed fracture surfaces of cemented tailings specimens after flexural tests under different cement–sand ratios and curing ages, following the same immersion duration. Using specimens with a 24 h immersion time as a unified benchmark, and maintaining a cement–sand ratio of 1:6 and 28-day curing age as the baseline condition, individual variables were systematically altered for comparative analysis. The water immersion patterns observed across different specimen groups are presented in Figure 7.

As shown in Figure 7, the water penetration depth in cemented tailings specimens exhibits a negative correlation with both the cement–sand ratio and curing age. As the cement–sand ratio decreased from 1:4 to 1:10, the proportion of cementitious material in the specimens correspondingly reduced, resulting in increased internal porosity. This led to greater water penetration depth under identical immersion conditions, progressively increasing from approximately 5 mm at a 1:4 ratio to 15.5 mm at a 1:8 ratio, until complete saturation occurred at the 1:10 ratio. With extended curing age from 7 to 28 days, more complete cement hydration developed increasingly dense microstructures, consequently reducing the penetration depth from complete saturation at 7 days to approximately 11 mm at 28 days. In summary, both increased cement–sand ratio and prolonged curing age promote microstructural densification in cemented tailings, which aligns with previous findings.

#### 3.2.2. Failure Morphology of Specimens After Uniaxial Compressive Tests

**Figure 8:**
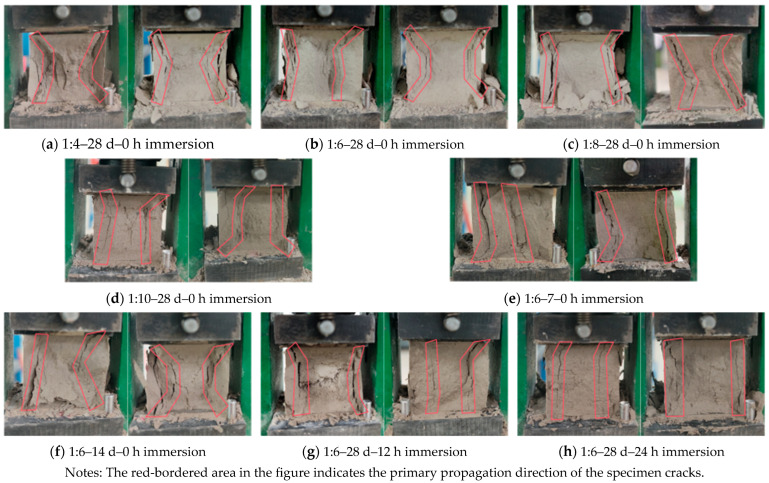
Failure modes of specimens under uniaxial compression.

Failure morphology serves as the external manifestation of internal damage evolution. By examining macroscopic characteristics such as crack propagation paths and fracture surface topography, it becomes possible to analyze the evolution of internal structures in cemented tailings under varying conditions, thereby correlating with mechanical performance test data [42]. The failure morphology of cemented tailings specimens exhibits notable variations under different influencing factors. Using specimens with a cement–sand ratio of 1:6, 28-day curing age, and 0 h immersion time as the baseline, individual variables were systematically modified for comparative analysis. Two representative specimens from each group were selected to characterize their failure modes, with results presented in Figure 8. Based on Figure 7, a mathematical characterization of the failure morphology of the cemented tailings specimens under uniaxial compression was conducted, with the results summarized in Table 3.

Under uniaxial compression, the failure of cemented tailings is predominantly manifested as tensile failure and shear failure. Shear failure is characterized by the absence of significant tensile cracks near the free surface of the fracture plane [43,44]. Based on the data presented in Table 2 and Table 3 and Figure 7, a clear correlation is observed between the failure modes and compressive strength of non-immersed specimens. When the compressive strength was relatively high (Specimens a and b), the dominant failure mode was conjugate inclined shear failure, characterized by intersecting diagonal cracks extending along the direction of maximum shear stress in an X-shaped pattern. These specimens exhibited larger angles between the major cracks and the symmetry axis (42°, 27°), longer crack lengths (44.92 mm, 41.61 mm), and wider crack openings (1.46 mm, 1.06 mm). As the compressive strength decreased (Specimens c and f), the crack inclination angles relative to the symmetry axis (25°, 36°), crack lengths (40.59 mm, 38.18 mm), and crack widths (0.60 mm, 0.71 mm) all diminished. The geometry of the conjugate fractures underwent a significant transformation—the curvature radius of the cracks increased, and the minimum spacing between the two main cracks widened. The failure mode transitioned to tensile-shear composite failure, exhibiting combined characteristics of diagonal shearing and vertical splitting. With further strength reduction (Specimens d and e), the failure mode shifted entirely to tension-dominated failure. This was marked by significantly reduced crack angles (9°, 11°), accompanied by decreased crack lengths (36.10 mm, 37.86 mm) and widths (0.43 mm, 0.64 mm), along with the development of nearly vertical cracks aligned parallel to the loading direction. Notably, although immersed, Specimens g and h maintained relatively high compressive strength, water-induced degradation of the internal microstructure resulted in a failure mode dominated by tensile-shear composite behavior, with crack angles sharply reduced to 2° and 4°.

### 3.3. Analysis of Strength Formation Mechanism in Cemented Tailings

#### 3.3.1. XRD Analysis

**Figure 9:**
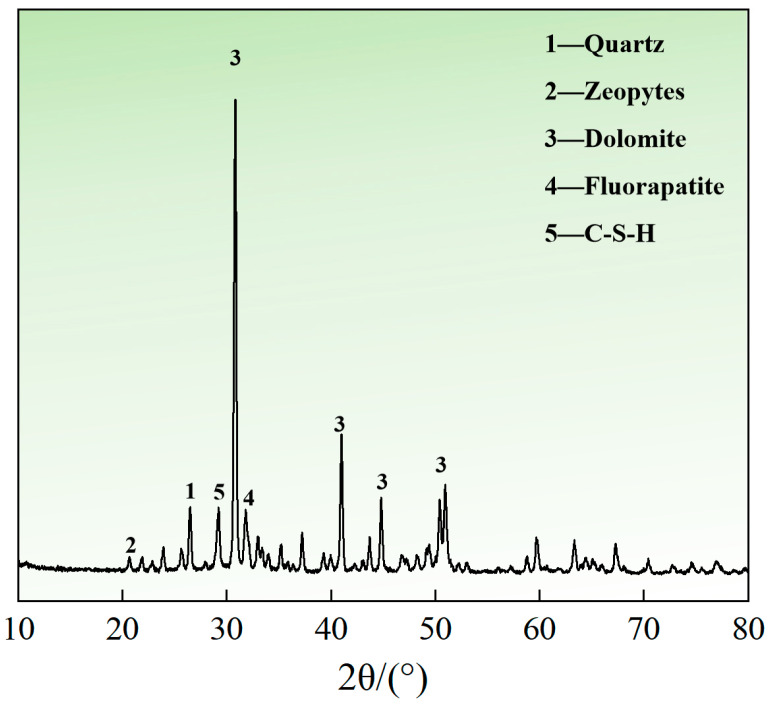
XRD pattern of cemented tailings.

As indicated in Table 2, the cemented tailings reached their optimal mechanical performance under natural curing conditions with a cement–sand ratio of 1:4 and a 28-day curing period. Therefore, specimens with 0 h immersion time (natural curing) under these conditions were selected for XRD analysis, with results presented in Figure 9. The pattern shows a broad diffuse hump primarily within the 25–55° range. The phase composition of this cemented tailings system is dominated by dolomite, accompanied by quartz and fluorapatite, while the hydration products mainly consist of C-S-H gel and zeotypes. Specifically, the diffuse peak observed near 29° confirms the formation of calcium silicate hydrate (C-S-H) gel, which serves as the primary strength-contributing phase in the cementitious system. The identification of dolomite as the predominant crystalline component was established through matching multiple characteristic diffraction peaks at 31° and 41° with standard reference patterns [45,46].

#### 3.3.2. SEM-EDS Analysis

**Figure 10:**
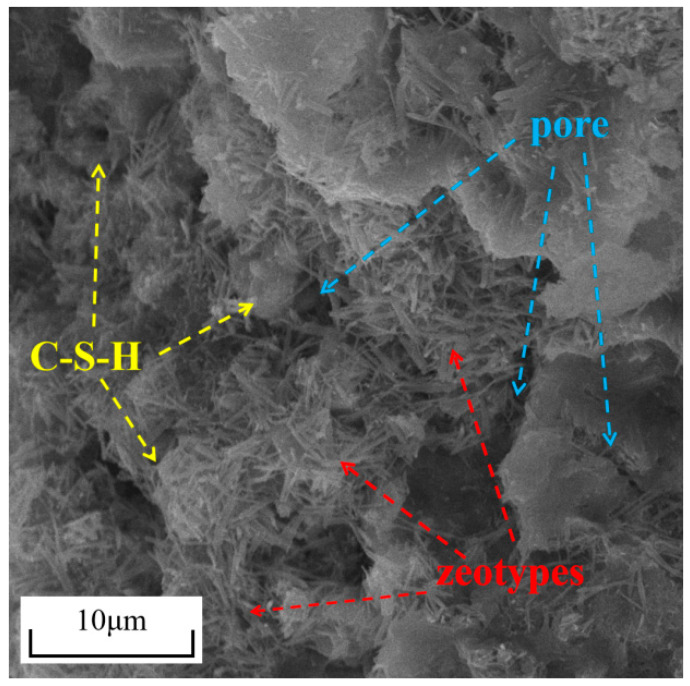
SEM image of cemented tailings.

Figure 10 presents the SEM images of cemented tailings with a cement–sand ratio of 1:4, 28-day curing age, and 0 h immersion time. The images reveal abundant acicular rod-shaped zeotypes and flocculent C-S-H gel formed in the cemented tailings. The zeotypes exhibit their characteristic acicular rod-like morphology, constructing a rigid crystalline skeleton network. Meanwhile, the amorphous C-S-H gel, with its highly developed specific surface area and dense flocculent structure, dispersively fills the pores within the system. These two products demonstrate both spatial independence and interpenetrative coexistence, collectively forming a highly continuous and robust integrated network in three-dimensional space. This microstructure effectively restrains plastic deformation and crack propagation in the material, thereby enhancing the structural integrity of the cemented tailings and ensuring its favorable mechanical properties.

**Figure 11:**
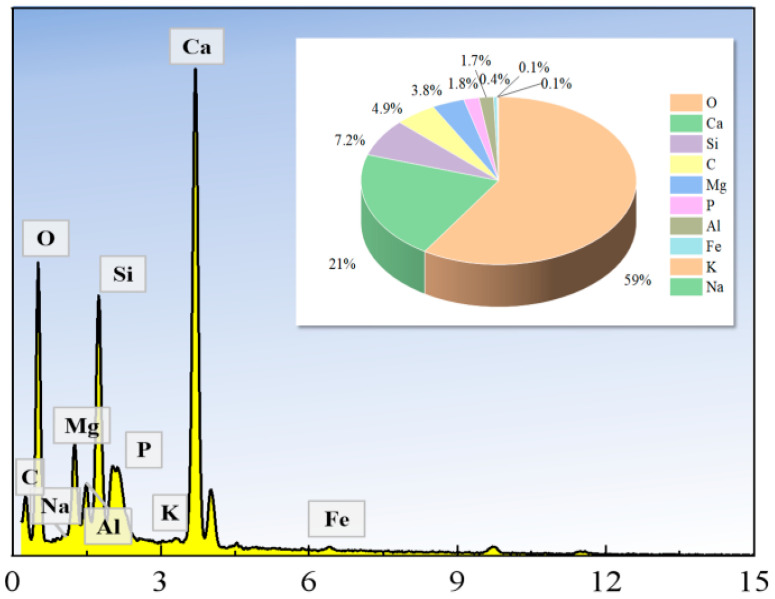
Elemental Composition Analysis of Cemented Tailings.

Figure 11 demonstrates that the primary elements present in the cemented tailings are O, Ca, Si, C, Mg, P, and Al. Among these, oxygen and calcium exhibit the highest mass fractions, reaching 59% and 21%, respectively. Combined with the characteristics of the cementitious material and the XRD results shown in Figure 9, it can be inferred that the cementitious system of the cemented tailings comprises a composite system where C-S-H gel coexists with zeotypes and other products, which aligns with the findings from the SEM analysis.

**Figure 12:**
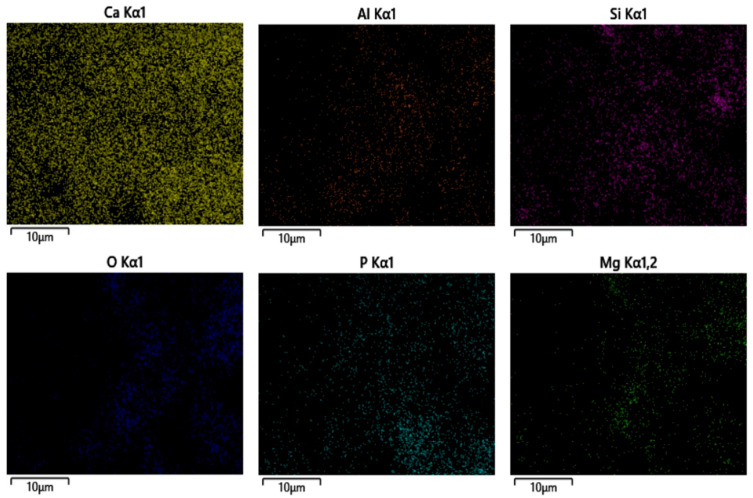
Surface element distribution of cemented tailings.

Figure 12 presents the EDS elemental mapping performed on the bulk surface of the cemented tailings, showing the distribution of six elements including Ca, Si, and Al. The results indicate a relatively uniform distribution of these elements within the cemented tailings matrix, with minimal localized enrichment or depletion zones. This homogeneous elemental distribution suggests substantially progressed hydration reactions throughout the tailings base material. The formation of primary hydration products, particularly zeotypes and C-S-H gel, demonstrates satisfactory prevalence and uniformity, ensuring the development of a continuous, homogeneous, and mechanically stable microstructure within the cemented tailings.

## 4. Discussion

### 4.1. Significance Analysis

To investigate the hierarchical influence and statistical significance of mass concentration, cement–sand ratio, moisture content, and curing age on the flexural and compressive strengths of cemented tailings, systematic analyses were performed using range analysis and ANOVA based on the experimental results in Table 2. Range analysis was applied to determine the primary-secondary order of the influencing factors, while ANOVA was used to examine the statistical significance of each factor’s effect. The results of the range analysis and ANOVA are presented in Table 4 and Table 5, respectively.

As shown in Table 4, the influencing factors demonstrate variation in their effects on flexural strength and compressive strength. Analysis of the flexural and compressive strength test results reveals that the *R*^2^ values of the factors follow the order A > B > C. This indicates that the cement–sand ratio exerted the most substantial influence on both the flexural and compressive strengths of the cemented tailings, followed by the curing age, while the specimen immersion time exerted the least influence.

Table 5 demonstrates that the cement–sand ratio, curing age, and specimen immersion time all exhibit statistically significant effects on both the flexural and compressive strengths of the cemented tailings (*p* < 0.001). The cement–sand ratio (*η*^2^ = 0.929 for flexural strength, *η*^2^ = 0.909 for compressive strength) and curing age (*η*^2^ = 0.806 for flexural strength, *η*^2^ = 0.851 for compressive strength) demonstrate the most substantial dominant effects. Although the specimen immersion time also shows a significant influence, its effect size is relatively smaller (*η*^2^ = 0.597 for flexural strength, *η*^2^ = 0.692 for compressive strength). The models exhibit strong overall explanatory power (*R*^2^ = 0.949 for flexural strength, *R*^2^ = 0.947 for compressive strength), confirming that the cement–sand ratio and curing age are the core parameters determining mechanical strength, while the specimen immersion time serves as an auxiliary regulating factor. These findings are consistent with the results of the range analysis.

### 4.2. Analytical Model for Strength Characteristics of Cemented Tailings

#### 4.2.1. Regression Model Development

Polynomial regression is widely utilized to develop mathematical models that capture both global and local effects of predictor variables on response variables, owing to its interpretable model structure and modeling flexibility [47]. Quadratic polynomial nonlinear regression effectively models constrained lower and upper bound effects, accommodates local linearity, and characterizes interaction effects along with nonlinear features [48]. When quadratic term coefficients approach zero, the model simplifies to linear regression. Employing quadratic polynomials as regression models therefore proves advantageous for investigating how variable factors influence the performance of cemented tailings. Using cement–sand ratio, curing age, and specimen immersion time as independent variables, with flexural strength and compressive strength as dependent variables, a mathematical model based on ternary quadratic polynomial nonlinear regression was constructed:

Based on the experimental results presented in Table 2, the relationships between flexural strength (*Y*_1_), compressive strength (*Y*_2_), and their respective independent variables were fitted. The regression coefficients for the two equations were statistically evaluated, with the significance statistics of these coefficients provided in Table 6.

The analysis results indicate that for both flexural and compressive strength, factors *A* (b) and *B* (c) exhibit significant (*p* < 0.01) positive effects. The interaction term between *A* and *B* (e) is significant in both strength models (*p* < 0.001 and *p* < 0.01, respectively), demonstrating a stable synergistic enhancement effect between them. Regarding nonlinear effects, the *A*^2^ (h) and *B*^2^ (i) terms in the compressive strength model are significant, indicating a notable curvilinear relationship in their influence, a phenomenon not observed in the flexural strength model. Furthermore, all terms related to factor *C* and some interaction terms (such as f, g, j) are not significant (*p* > 0.05), suggesting their minimal impact within the selected experimental parameter range. These findings are consistent with the significance analysis presented earlier.

Based on the estimated values of each parameter in Table 6, the multivariate nonlinear regression equations for flexural strength and compressive strength were integrated and summarized, with the results presented in Table 7.

In regression analysis, the *F*-statistic and *R*^2^ (coefficient of determination) are key parameters for evaluating a model’s explanatory power. The F-statistic tests the overall significance of the model, assessing whether the combined effect of all independent variables on the dependent variable is statistically significant. A larger *F* with a corresponding *p* < 0.05 indicates that the model significantly outperforms a null model containing only an intercept, confirming that at least one independent variable effectively explains variation in the dependent variable. *R*^2^ quantifies the proportion of variance in the dependent variable explained by the model (ranging from 0 to 1). Values closer to 1 indicate better model fit. As shown in Table 7, all Fs exceed the critical value *F*_0.01_(11,25) ≈ 3.18, demonstrating that the overall models are statistically significant. The values for *Y*_1_ and *Y*_2_ are 0.961 and 0.976, respectively, indicating that the regression equations explain 96.1% and 97.6% of the variance in their corresponding dependent variables, confirming excellent model fit.

#### 4.2.2. Regression Model Validation

To validate the equations derived in Table 7, a new set of cemented tailings specimens was prepared and subjected to flexural and compressive strength tests. The experimental results were compared with the predicted values generated by the models in Table 7, with the comparative outcomes presented in Table 8.

Analysis of Table 8 shows that the absolute error range for flexural strength is 3.90–9.52%, with a mean error of 7.25%, while the absolute error range for compressive strength is 4.16–9.27%, with a mean error of 5.81%. All relative errors for the evaluated parameters remain below 10%, and the overall mean error is 6.53%, demonstrating close agreement between the predicted and experimental values.

## 5. Conclusions

(1) Through 36 comprehensive experiments considering cement–sand ratio, curing age, and specimen immersion time as key factors, the flexural strength of cemented tailings ranged from 0.43 to 2.07 MPa and compressive strength from 3.02 to 12.52 MPa within the selected parameter ranges. Both strength parameters reached their maximum values at a cement–sand ratio of 1:4, 28-day curing age, and 0 h immersion time.
(2) Within the experimental scope, both cement–sand ratio and curing age demonstrated positive correlations with the strength of cemented tailings specimens. The 24 h water penetration depth exhibited consistent variation patterns with strength development. The compressive failure mode transitioned from conjugate shear failure (X-shaped cracks) to tensile-shear composite failure as strength decreased, eventually evolving into tension-dominated failure.
(3) The primary hydration products in cemented tailings consisted of flocculent C-S-H and acicular zeotypes. The rigid zeotype framework embedded and penetrated the dense C-S-H gel matrix, while the gel phase tightly encapsulated and cemented the zeotype crystals. These two phases formed a homogeneous interpenetrating structure throughout the cemented tailings, collaboratively constructing a three-dimensional network with favorable mechanical properties.
(4) The order of significance for both flexural and compressive strength influences was cement–sand ratio > curing age > specimen immersion time. All three factors significantly affected the mechanical strengths of cemented tailings, with cement–sand ratio and curing age identified as the core parameters determining mechanical strength, while specimen immersion time served as an auxiliary regulating factor.
(5) Comprehensive predictive mathematical models based on ternary quadratic polynomials were established to relate the factors to strength indicators of cemented tailings. Validation through five experimental tests showed all relative errors below 10%, with a mean error of 6.53%. The models demonstrated high accuracy within the selected variable ranges.

## Figures and Tables

**Table 1:** Physical properties and composition of tailings and cementitious materials.

Materials	Physical Properties of Materials				Mass Fraction of Each Component/%						
	Bulk Density /(g·cm^−3^)	Density /(g·cm^−3^)	Compactness /%	Porosity /%	MgO	Al_2_O_3_	SiO_2_	P_2_O_5_	CaO	Fe_2_O_3_	Other
Tailings	1.47	1.72	85.47	14.53	18.71	1.97	8.19	8.65	45.27	1.86	15.34
Cementitious material	1.06	1.35	78.33	21.67	5.97	17.38	24.92	0.19	40.16	2.12	9.26

**Table 2:** Mechanical Properties Test Results of Cemented Tailings.

Cement–Sand Ratio	Specimen Immersion Time	7 d			14 d			28 d		
		Average Mass of Test Specimens (g)	Flexural Strength (MPa)	Compressive Strength (MPa)	Average Mass of Test Specimens (g)	Flexural Strength (MPa)	Compressive Strength (MPa)	Average Mass of Test Specimens (g)	Flexural Strength (MPa)	Compressive Strength (MPa)
1:4	0 h	446.55	1.31 ± 0.06	8.95 ± 0.39	456.25	1.89 ± 0.16	10.77 ± 1.05	444.15	2.07 ± 0.12	12.52 ± 0.71
	12 h	493.00	1.24 ± 0.14	6.76 ± 0.81	491.1	1.41 ± 0.08	9.63 ± 0.52	490.85	1.85 ± 0.10	11.03 ± 0.72
	24 h	508.40	1.07 ± 0.03	5.64 ± 0.19	499.1	1.29 ± 0.11	8.19 ± 0.77	495.60	1.69 ± 0.05	10.27 ± 0.27
1:6	0 h	432.80	0.88 ± 0.02	6.55 ± 0.08	433.10	1.25 ± 0.09	8.93 ± 0.89	426.95	1.65 ± 0.19	11.25 ± 1.39
	12 h	486.50	0.66 ± 0.04	4.94 ± 0.30	485.55	1.04 ± 0.12	7.00 ± 0.79	489.15	1.32 ± 0.06	10.00 ± 0.43
	24 h	504.35	0.58 ± 0.04	3.89 ± 0.23	503.45	0.76 ± 0.07	6.58 ± 0.49	501.25	1.19 ± 0.10	9.06 ± 0.92
1:8	0 h	450.45	0.59 ± 0.03	4.44 ± 0.14	436.05	0.87 ± 0.08	6.94 ± 0.71	440.40	1.07 ± 0.09	7.80 ± 0.82
	12 h	487.95	0.53 ± 0.05	3.78 ± 0.38	493.6	0.72 ± 0.06	5.66 ± 0.41	488.10	0.92 ± 0.05	6.15 ± 0.43
	24 h	505.15	0.42 ± 0.02	3.67 ± 0.04	508.1	0.64 ± 0.09	4.16 ± 0.67	506.85	0.88 ± 0.06	5.28 ± 0.30
1:10	0 h	436.50	0.50 ± 0.03	3.68 ± 0.26	425.85	0.71 ± 0.03	5.92 ± 0.28	433.35	0.82 ± 0.07	6.39 ± 0.53
	12 h	491.30	0.44 ± 0.02	3.25 ± 0.07	493.4	0.58 ± 0.02	5.00 ± 0.24	491.95	0.65 ± 0.04	5.48 ± 0.33
	24 h	500.95	0.43 ± 0.04	3.02 ± 0.20	504.35	0.49 ± 0.08	3.34 ± 0.81	509.40	0.59 ± 0.03	4.28 ± 0.17

**Table 3:** Analysis of Mathematical Characteristics of Specimen Failure Morphology.

Specimen	Inclination Angle of the Major Crack Relative to the Symmetry Axis	Maximum Crack Length
(a) 1:4–28 d–0 h immersion	42°	44.92 mm
(b) 1:6–28 d–0 h immersion	27°	41.61 mm
(c) 1:8–28 d–0 h immersion	25°	40.59 mm
(d) 1:10–28 d–0 h immersion	9°	36.10 mm
(e) 1:6–7–0 h immersion	11°	37.86 mm
(f) 1:6–14 d–0 h immersion	36°	38.18 mm
(g) 1:6–28 d–12 h immersion	4°	38.05 mm
(h) 1:6–28 d–24 h immersion	2°	35.61 mm

**Table 4:** Range analysis table.

Factor	Flexural Strength			Compressive Strength		
	Cement–Sand Ratio A	Curing Age B	Specimen Immersion Time C	Cement–Sand Ratio A	Curing Age B	Specimen Immersion Time C
K_avg_	1.53	0.72	1.13	9.35	4.88	7.84
	1.04	0.97	0.95	7.58	6.85	6.62
	0.74	1.22	0.84	5.35	8.38	5.65
	0.58	-	-	4.54	-	-
*R*	0.96	0.50	0.30	4.81	3.50	2.20
*R* ^2^	1.29	0.91	0.54	6.49	6.31	3.96

Notes: K_avg_ represents the mean response value (average flexural/compressive strength) at each factor level; *R* (range) reflects the difference between the maximum and minimum values across factor levels; *R*^2^ (contribution rate) quantifies the proportional influence of each factor on the total variation.

**Table 5:** ANOVA table.

Factor	Flexural Strength			Compressive Strength		
	*F*	*p*	*η* ^2^	*F*	*p*	*η* ^2^
Intercept	2610.767	*** *p* < 0.001	0.989	3504.064	*** *p* < 0.001	0.992
Cement–sand ratio A	122.015	*** *p* < 0.001	0.929	92.727	*** *p* < 0.001	0.909
Curing age B	58.128	*** *p* < 0.001	0.806	80.125	*** *p* < 0.001	0.851
Specimen immersion time C	20.750	*** *p* < 0.001	0.597	31.446	*** *p* < 0.001	0.692
*R* ^2^	0.949			0.947		
Adjusted *R*^2^	0.937			0.934		

Note: *F* represents the ratio of between-group variation to within-group variation; *η*^2^ (partial eta-squared) measures the proportion of variance independently explained by each factor; the *p* serves as the significance threshold for determining statistical significance, where asterisks denote statistical significance: *** *p* < 0.001 indicates highly significant effects.

**Table 6:** Significance Statistics of Regression Coefficients.

Parameter	Flexural Strength				Compressive Strength			
	Estimate	Standard Error	*t*	*p*	Estimate	Standard Error	*t*	*p*
a	−0.453	0.207	−2.188	* *p* < 0.05	−4.643	1.546	−3.003	** *p* < 0.01
b	6.960	2.152	3.234	** *p* < 0.01	74.467	16.072	4.633	*** *p* < 0.001
c	0.035	0.012	2.917	** *p* < 0.01	0.347	0.088	3.943	*** *p* < 0.001
d	−0.005	0.007	−0.714	*p* > 0.05	−0.072	0.051	−1.412	*p* > 0.05
e	0.123	0.027	4.556	*** *p* < 0.001	0.691	0.202	3.421	** *p* < 0.01
f	−0.070	0.024	−2.917	** *p* < 0.01	−0.215	0.180	−1.194	*p* > 0.05
g	0.000	0.000	-	*p* > 0.05	0.000	0.001	0.000	*p* > 0.05
h	−4.904	5.824	−0.842	*p* > 0.05	−142.473	43.507	−3.275	** *p* < 0.01
i	−0.001	0.000	-	*p* > 0.05	−0.008	0.002	−4.000	*** *p* < 0.001
j	0.000	0.000	-	*p* > 0.05	0.001	0.001	1.000	*p* > 0.05

Note: Parameters are defined based on the model: *Y* = a + b*A* + c*B* + d*C* + e*AB* + f*AC* + g*BC* + h*A*^2^ + i*B*^2^ + j*C*^2^. The *t* measures the reliability of the coefficient estimate, with a larger absolute value indicating a more significant effect of the variable; the *p* represents the probability used to test for statistical significance, where * *p* < 0.05 indicates significance, ** *p* < 0.01 indicates strong significance, and *** *p* < 0.001 indicates highly significant effects.

**Table 7:** Multivariate Nonlinear Regression Results.

Mechanical Properties	Regression Equation		*F*-Statistic	*R* ^2^
Flexural strength	$Y_{1}=-0.452+6.960A+0.040B+0.005C+0.123\mathrm{AB}-0.070\mathrm{AC}-4.90A^{2}-0.001B^{2}$	(3)	508.50	0.961
Compressive strength	$Y_{2}=-4.643+74.466A+0.347B-0.072C+0.691\mathrm{AB}-0.215\mathrm{AC}-142.473A^{2}-0.008B^{2}+0.001C^{2}$	(4)	631.54	0.976

**Table 8:** Regression model validation results.

No.	Cement–Sand Ratio	Curing Age (d)	Specimen Immersion Time (h)	Predicted Flexural Strength (MPa)	Predicted Compressive Strength (MPa)	Experimental Flexural Strength (MPa)	Experimental Compressive Strength (MPa)	Absolute Error of Flexural Strength	Absolute Error of Compressive Strength
1	0.40 (1:2.5)	14	0	2.56	9.47	2.77	8.98	7.42	5.43
2	0.25 (1:4)	7	6	1.27	7.58	1.22	6.94	3.90	9.27
3	0.30 (1:3.33)	14	12	1.73	9.52	1.62	9.93	6.84	4.16
4	0.10 (1:10)	28	18	0.66	5.21	0.73	5.46	9.52	4.64
5	0.20 (1:5)	28	24	1.32	9.46	1.48	10.01	8.59	5.54

## Data Availability

The original contributions presented in this study are included in the article. Further inquiries can be directed to the corresponding author.

## Abbreviations

The following abbreviations are used in this manuscript:

C-S-H	Calcium silicate hydrate
XRD	X-ray diffraction
SEM	Scanning electron microscope
EDS	Energy dispersive spectrometer
ANOVA	Analysis of variance
